# Editorial: Ion transporters and channels in cellular pathophysiology

**DOI:** 10.3389/fcell.2022.1049433

**Published:** 2022-10-17

**Authors:** Alessia Remigante, Paola Gavazzo, Rossana Morabito, Silvia Dossena

**Affiliations:** ^1^ Department of Chemical, Biological, Pharmaceutical and Environmental Sciences, University of Messina, Messina, Italy; ^2^ Biophysics Institute, National Research Council, Genova, Italy; ^3^ Institute of Pharmacology and Toxicology, Paracelsus Medical University, Salzburg, Austria

**Keywords:** ion channels, ion transporters, disease, kidney, heart, erythrocytes

Ion and water channels and transporters mediate the flux of ions, water, and small- and medium-size molecules across cellular membranes in response to specific stimuli. Ion transport is crucial for proper cell, tissue, and organ function, including the regulation of cardiac and neuronal activity, systemic pH, salt and water homeostasis, as well as cell proliferation, differentiation, and apoptosis. As a consequence, the impairment of any of its molecular components may have dramatic consequences for cellular and organ physiology, and eventually for human health. Therefore, the precise understanding of the mechanisms of physiological regulation and pathological dysregulation of ion transport is essential to open new avenues in therapy and drug design. The purpose of this Research Topic was to collect and contribute to the dissemination of high-quality research articles summarizing the current progress in the understanding of different aspects of ion channels and transporters, including intracellular signaling. The 10 original publications collected are well representative of the variety of roles of ion channels and transporters and highlight the multitude of the experimental models and approaches currently used to unravel their complexity. Hereafter we offer an overview of the content of the Research Topic.

Alterations in the ion transport can cause serious diseases affecting the heart and the central nervous system. Among these, sudden cardiac arrest (SCA) is a global public health problem. Most cases of SCA are caused by cardiac arrhythmias that may arise from functional changes of ion channels that underlie the cardiac action potential (AP) and can be evoked by various drugs used for the treatment of cardiac or non-cardiac conditions, including carbamazepine (CBZ). In their manuscript, Jia et al. showed a correlation between CBZ treatment and SCA and demonstrated that CBZ reduced the AP upstroke velocity by reducing the cardiac Na^+^ current at therapeutic concentrations and shortened the AP duration by reducing the L-type Ca^2+^ currents at high concentrations. Thus, these data mechanistically explain the increase of SCA risk upon CBZ exposure (Jia et al.).

Pathogenic sequence alterations in the *LMNA* gene coding for the type-V intermediate filament proteins Lamin A/C are linked to muscular dystrophies and cardiac abnormalities associated with fatal arrhythmias. De Zio et al. found that cardiomyocytes expressing the truncated Lamin A/C Q517X variant exhibit action potential abnormalities, tubulin hyper-polymerization and hyper-acetylation, and defective cell surface Nav1.5 expression. The authors provided evidence showing that pharmacological manipulation of tubulin state might represent a novel strategy in the treatment of cardiac laminopathies (De Zio et al.).

The Ca^2+^-releasing second messenger nicotinic acid adenine dinucleotide phosphate (NAADP) mobilizes lysosomal Ca^2+^ through two-pore channels (TPCs), thereby modulating the excitation-contraction (EC) coupling in vascular smooth muscle cells and cardiomyocytes and driving the angiogenic behaviour of vascular endothelial and endothelial progenitor cells (Moccia et al., 2021). Faris et al. have examined the Ca^2+^ signalling in cardiac mesenchymal stromal cells (C-MSCs) and revealed that the NAADP-induced lysosomal Ca^2+^ release stimulates inositol-1,4,5-trisphosphate (InsP_3_)-induced Ca^2+^ mobilization from the endoplasmic reticulum (ER) through the mechanism of Ca^2+^-induced Ca^2+^ release. The functional interaction between acidic and neutral Ca^2+^ stores and activation of TPCs permits the C-MSC proliferation induced by extracellular signals. This pathway might offer novel therapeutic targets in cardiac repair (Faris et al.).

The role of intracellular calcium in non-excitable cells including fibroblasts is incompletely understood. Histamine could act as an inflammatory mediator in asthmatic lungs by stimulating proliferation and migration of resident fibroblasts through an increase in [Ca^2+^]_i_. Berra-Romani et al. investigated the mechanism whereby histamine induces intracellular Ca^2+^ signals in the human lung fibroblast cell line WI-38 and found that these signals are triggered by histamine one receptors (H1Rs) and partially contributed by H2Rs (Berra-Romani et al.). The Ca^2+^ response is mediated by InsP_3_-induced Ca^2+^ release from the ER and maintained over time by store-operated Ca^2+^ entry. The authors suggest that pharmacological blockade of InsP_3_ receptors and store-operated channels could represent an alternative strategy to prevent the deleterious effects of histamine on lung fibroblasts in asthmatic patients.

The K^+^-Cl^−^ cotransporter KCC2 is expressed in neurons throughout the central nervous system and deficits in its activity have been implicated in a variety of neurological disorders, including epilepsy. Prael III et al. discovered and characterized a new class of small-molecule KCC2 potentiators and showed that pharmacological KCC2 potentiation, by itself, is sufficient to attenuate neuronal hyperexcitability in an *in vitro* model of cortical neuronal-glial co-cultures that is sensitive to anti-epileptic drugs (Prael et al.). These results pave the way to the development of KCC2-directed therapeutics for multiple neurological disorders.

Yap et al. characterized a *Dictyostelium discoideum* model of CLN7 disease, which is a form of neurodegeneration in humans linked to mutations in *MFSD8* (Yap et al.). The MFSD8 protein has been reported to transport chloride ions across the lysosomal membrane and, in their study, Yap et al. showed that the *D. discoideum* homolog of MFSD8 plays an important role in modulating a variety of cellular processes during the growth and early development of the organism.

Ion transport in the kidney is fundamental in the regulation of systemic pH and blood pressure. Ferdaus et al. show that the potassium-chloride co-transporter KCC3a co-localizes with the chloride/bicarbonate exchanger pendrin (SLC26A4) in the apical membrane of type B and non-A/non-B intercalated cells in the mouse kidney cortex (Ferdaus et al.). As metabolic alkalosis upregulated both pendrin and KCC3a, the authors suggested that KCC3a might provide a pathway for recycling of chloride ions across the apical membrane to permit bicarbonate secretion *via* pendrin. A functional interplay between these two transporters could explain the mechanism of hypokalemia in metabolic alkalosis.

Xu et al. identified the IQ motif-containing GTPase-activating protein 1 (IQGAP1) as a protein that binds to the C-terminus of pendrin and co-localizes with pendrin on the apical membrane of B-intercalated cells in the mouse kidney. Functional studies in cell-based systems demonstrated that IQGAP1 favors the plasma membrane localization of pendrin and enhances its Cl^−^/HCO_3_^−^ exchanger activity. The authors suggested that a disturbed interaction between these two proteins might contribute to pathophysiological states characterized by an altered function or trafficking of pendrin (Xu et al.).

In addition to kidneys, also the colonic epithelium, which expresses the water channel AQP3, modulates water and salt homeostasis. Centrone et al. evaluated the action of vasopressin (AVP) on the AQP3 expression and function by using selective vasopressin receptors (VRs) antagonists in human colonic HCT8 cells (Centrone et al.). dDAVP reduced AQP3 activity and cell viability in a V1aR-dependent, V2R-independent manner. Interestingly, in the colon of patients with adenocarcinoma, the expression of V1aR was significantly decreased. These findings identify the AVP-dependent AQP3 pathway as a novel target in colon diseases associated with abnormal cell growth.

Ferreira et al. characterized the regulation of red blood cells (RBC) Mg^2+^ in conditions of hyperglycemia (Ferreira et al.). Exposure of RBC to 50 mM d-glucose lowered cellular Mg^2+^ and enhanced the Na^+^/Mg^2+^ exchange activity. Similar changes were also observed in RBC from individuals with type 2 diabetes (T2D) or a diabetic mouse model and were reversed by reducing the N-glycosylation state of membrane proteins. These data show that the decreased Mg^2+^ content of RBC in T2D reflects enhanced Na^+^/Mg^2+^ exchange and further suggest that N-deglycosylation of targets within the pathway of Mg^2+^ regulation may have therapeutic potential in treatment of the vascular complications of T2D.

To conclude, the precise understanding of the ion transport pathophysiology is predicted to favor the identification of novel therapeutic targets and strategies for conditions ranging from cardiac diseases, epilepsy, asthma, and T2D.
